# An Ensemble Learning Approach to Improving Prediction of Case Duration for Spine Surgery: Algorithm Development and Validation

**DOI:** 10.2196/39650

**Published:** 2023-01-26

**Authors:** Rodney Allanigue Gabriel, Bhavya Harjai, Sierra Simpson, Austin Liu Du, Jeffrey Logan Tully, Olivier George, Ruth Waterman

**Affiliations:** 1 Division of Biomedical Informatics Department of Medicine University of California, San Diego San Diego, CA United States; 2 Division of Perioperative Informatics Department of Anesthesiology University of California, San Diego San Diego, CA United States; 3 School of Medicine University of California, San Diego San Diego, CA United States; 4 Department of Psychiatry University of California, San Diego San Diego, CA United States; 5 Department of Anesthesiology University of California, San Diego San Diego, CA United States

**Keywords:** ensemble learning, machine learning, spine surgery, case duration, prediction accuracy, operating room efficiency, learning, surgery, spine, operating room, case, model, patient, surgeon, linear regression, accuracy, estimation, time

## Abstract

**Background:**

Estimating surgical case duration accurately is an important operating room efficiency metric. Current predictive techniques in spine surgery include less sophisticated approaches such as classical multivariable statistical models. Machine learning approaches have been used to predict outcomes such as length of stay and time returning to normal work, but have not been focused on case duration.

**Objective:**

The primary objective of this 4-year, single-academic-center, retrospective study was to use an ensemble learning approach that may improve the accuracy of scheduled case duration for spine surgery. The primary outcome measure was case duration.

**Methods:**

We compared machine learning models using surgical and patient features to our institutional method, which used historic averages and surgeon adjustments as needed. We implemented multivariable linear regression, random forest, bagging, and XGBoost (Extreme Gradient Boosting) and calculated the average *R*^2^, root-mean-square error (RMSE), explained variance, and mean absolute error (MAE) using k-fold cross-validation. We then used the SHAP (Shapley Additive Explanations) explainer model to determine feature importance.

**Results:**

A total of 3189 patients who underwent spine surgery were included. The institution’s current method of predicting case times has a very poor coefficient of determination with actual times (*R*^2^=0.213). On k-fold cross-validation, the linear regression model had an explained variance score of 0.345, an *R*^2^ of 0.34, an RMSE of 162.84 minutes, and an MAE of 127.22 minutes. Among all models, the XGBoost regressor performed the best with an explained variance score of 0.778, an *R*^2^ of 0.770, an RMSE of 92.95 minutes, and an MAE of 44.31 minutes. Based on SHAP analysis of the XGBoost regression, body mass index, spinal fusions, surgical procedure, and number of spine levels involved were the features with the most impact on the model.

**Conclusions:**

Using ensemble learning-based predictive models, specifically XGBoost regression, can improve the accuracy of the estimation of spine surgery times.

## Introduction

Surgery is an important component of care for many patients experiencing pathology of the spine. Lower back pain, degenerative disease of the spine, and other related ailments cost the United States tens of billions of dollars a year in direct medical expenses and lost productivity [1]. Martin and colleagues [2] reported the incidence of elective fusion of the lumbar spine increasing over 60% from 2004 to 2015, with hospital costs for such surgeries surging over 170% in the same time to an average of over US $50,000 per admission. Despite new trends in cost containment [3-5], new operative techniques, expansion of surgical navigation and imaging systems, implementation of specialized postoperative recovery pathways, and increased demand for services in an aging patient population have resulted in a complex, highly variable operational environment [6-9]. Such heterogeneity can make planning and use of resources challenging. The operating room is a critical target for decreasing costs and is second only to the patient room and board in the total expense of a perioperative episode [10]. Many strategies for improving operating room efficiency focus on time management [11,12]. Predicted surgical case duration often informs how cases are scheduled and which resources are dedicated to prepare for and staff them [13]. Consequently, improving the accuracy of these predictions is a practical strategy to increase operating room efficiency [14].

Surgeons often estimate case durations when scheduling operative time; durations may also be tied to historical averages or Current Procedural Terminology (CPT) codes, practices that are prone to substantial inaccuracies [15]. Classical statistical methods have been used to further improve the prediction of case durations [16-18]. The proliferation of electronic health records and the associated generation of vast amounts of previously uncaptured patient data have allowed for more sophisticated analytics in several clinical arenas, including the operating room [19]. With large enough data sets, specialized algorithms can develop complex predictive models after being exposed to a number of prior examples in a process known as machine learning [20].

Current predictive techniques in spine surgery include less sophisticated approaches such as classical multivariable statistical models. While a variety of features and outcomes, such as length of stay, prescription duration, and time to return to normal work, have been predicted in previous studies, there has been little focus on case duration [21-25]. To our knowledge, no other studies have focused on using machine learning models to predict the surgical case duration for the spine surgery population, but the method has been implemented in other procedures [26-28]. Spine surgery consists of heterogenous anatomical and technical components that should theoretically be taken into account when estimating case duration. The primary objective of this study is to develop machine learning-based predictive models using patient and surgery-specific features. Specifically, we use ensemble learning, which combines multiple predictive models to determine an overall prediction of the outcome. We hypothesize that such models can outperform those that estimate case duration based on historic averages and surgeon preference (which may not be scalable or transferable outside of a given institution).

## Methods

### Ethics Approval

This retrospective study was approved (approval protocol 210098) by the Human Research Protections Program at the University of California, San Diego for the collection of data from our electronic medical record system. For this study, the informed consent requirement was waived. Data were collected retrospectively from the electronic medical record system of our institution’s operating room data. Data from all patients that underwent spine surgery from 3 different orthopedic spine surgeons from January 2018 to September 2021 was extracted. We excluded all patients that had missing data for actual case duration; all other features with missing values were categorized as unknown or imputed if they were continuous variables (described below). This retrospective observational study abided by the EQUATOR guidelines.

### Primary Objective and Data Collection

The primary outcome measurement was a continuous value, defined as the actual operating room case duration measured in minutes (from patient wheeling into the operating room to exiting the operating room). We implemented predictive models using various machine learning algorithms to predict the actual case duration. We compared this to our current system’s practice of estimating case duration, which is equal to the mean of the last 3 times the surgical procedure was performed, with the ability of the surgeon to change times based on their preference. The models developed were multivariable linear regression, random forest regressors, bagging regressors, and XGBoost (Extreme Gradient Boosting) regressors.

The independent features in the models were (1) categorical features, which included surgical procedure (39 unique procedures), surgeon identification (3 different surgeons), American Society of Anesthesiologists Physical Status (ASA PS) score (ie, comorbidity burden), sex, specific surgical details (kyphoplasty, discectomy, fusion, and laminectomy), the anterior approach involved (ie, approach surgeon used to access the spine), and level of spine region involved (eg, cervical, thoracic, lumbar, or a combination of levels); and (2) continuous features, which included the number of spine levels involved in the surgery (from 1 to 7) and body mass index (kg/m^2^) (Table 1). For missing data on the ASA PS class, the value was defined as “unknown.” For missing data on body mass index, the value was imputed by using the average BMI among all patients with known data for this feature.

**Table 1:** Characteristics of all the cases included in analysis (N=3315).

Characteristics				Instrumentation		Approach		Fusion		Levels		Other		Participants, n (%)
**Surgical Procedure**														
	Discectomy			No		Anterior		Yes		1		Fusion		89 (2.7)
	Discectomy			No		Anterior		Yes		2		Fusion		127 (3.8)
	Discectomy			No		Anterior		Yes		3+		Fusion		202 (6.1)
	Deformity fusion			No		Posterior		Yes		1-6 seg.		For deformity		8 (0.2)
	Deformity fusion			No		Posterior		Yes		7-12 seg.		For deformity		2 (0.1)
	Lumbar fusion			No		Anterior		Yes		2		Lumbar		270 (8.1)
	Lumbar fusion			No		Anterior		Yes		3		Lumbar		14 (0.4)
	Oblique lumbar interbody fusion			No		Anterior		Yes		1		Lumbar		1 (0.0)
	Transforaminal lumbar interbody fusion			No		Transforaminal		Yes		1		Lumbar		9 (0.3)
	Extreme lateral interbody fusion			No		Lateral		Yes		1		Lumbar		251 (7.6)
	Extreme lateral interbody fusion			No		Lateral		Yes		2		Lumbar		198 (6.0)
	Extreme lateral interbody fusion			No		Lateral		Yes		3		Lumbar		63 (1.9)
	Extreme lateral interbody fusion			No		Lateral		Yes		4		Lumbar		16 (0.5)
	Thoracic fusion			No		Posterior		Yes		1		Thoracic		1 (0.0)
	Thoracic fusion			No		Posterior		Yes		2		Thoracic		7 (0.2)
	Thoracic fusion			No		Posterior		Yes		3		Thoracic		17 (0.5)
	Thoracic fusion			No		Posterior		Yes		4		Thoracic		12 (0.4)
	Thoracic fusion			No		Posterior		Yes		5+		Thoracic		35 (1.1)
	Kyphoplasty or vertebroplasty			No		N/A		No		1		All		316 (9.5)
	Kyphoplasty			No		N/A		No		2		Thoracolumbar		40 (1.2)
	Kyphoplasty			No		N/A		No		3		Thoracolumbar		19 (0.6)
	Kyphoplasty			No		N/A		No		4		Thoracolumbar		21 (0.6)
	Laminectomy or decompressive laminectomy			No		Posterior		No		1		Lumbar		148 (4.5)
	Laminectomy or decompressive laminectomy			No		Posterior		No		2		Lumbar		106 (3.2)
	Laminectomy or decompressive laminectomy			No		Posterior		No		3		Lumbar		110 (3.3)
	Laminectomy			No		Posterior		Yes		5		Cervical		109 (3.3)
	Laminectomy			No		Posterior		Yes		1-4		Cervical		115 (3.5)
	Laminectomy			No		Posterior		No		1-2		Cervical		3 (0.1)
	Laminectomy			No		Posterior		No		2+		Cervical		31 (0.9)
	Laminectomy			Yes		Posterior		Yes		1		Lumbar		219 (6.6)
	Laminectomy			Yes		Posterior		Yes		2		Lumbar		259 (7.8)
	Laminectomy			Yes		Posterior		Yes		3		Lumbar		162 (4.9)
	Laminectomy			Yes				Yes		4+		Lumbar		259 (7.8)
	Laminectomy			Yes		Posterior		Yes		1		Thoracic		9 (0.3)
	Laminectomy			Yes		Posterior		Yes		2		Thoracic		9 (0.3)
	Laminectomy			Yes		Posterior		Yes		3		Thoracic		7 (0.2)
	Laminectomy			Yes		Posterior		Yes		4		Thoracic		18 (0.5)
	Laminectomy			Yes		Posterior		Yes		5		Thoracic		30 (0.9)
	Laminectomy			Yes		Posterior		Yes		6+		Thoracic		3 (0.1)
**Specific surgical procedure included**														
		Kyphoplasty	N/A^a^		N/A		N/A		N/A		N/A		396 (11.9)	
		Discectomy	N/A		N/A		N/A		N/A		N/A		418 (12.6)	
		Fusion	N/A		N/A		N/A		N/A		N/A		2521 (76.0)	
		Laminectomy	N/A		N/A		N/A		N/A		N/A		1597 (48.2)	
Anterior approach involved				N/A		N/A		N/A		N/A		N/A		702 (21.1)
**Number of spine levels involved**														
		1	N/A		N/A		N/A		N/A		N/A		1043 (31.5)	
		2	N/A		N/A		N/A		N/A		N/A		1050 (31.7)	
		3	N/A		N/A		N/A		N/A		N/A		594 (17.9)	
		4	N/A		N/A		N/A		N/A		N/A		441 (13.3)	
		5	N/A		N/A		N/A		N/A		N/A		174 (5.2)	
		6	N/A		N/A		N/A		N/A		N/A		11 (0.3)	
		7	N/A		N/A		N/A		N/A		N/A		2 (0.1)	
**Surgeon performing procedure**														
		A	N/A		N/A		N/A		N/A		N/A		1676 (50.6)	
		B	N/A		N/A		N/A		N/A		N/A		191 (5.8)	
		C	N/A		N/A		N/A		N/A		N/A		1448 (43.7)	
**Level of spine involved**														
		Cervical	N/A		N/A		N/A		N/A		N/A		567(17.1)	
		Thoracic	N/A		N/A		N/A		N/A		N/A		228 (6.9)	
		Lumbar	N/A		N/A		N/A		N/A		N/A		2165 (65.3)	
Male sex				N/A		N/A		N/A		N/A		N/A		1800 (54.3)
BMI (kg/m^2^), mean (SD)				N/A		N/A		N/A		N/A		N/A		29.7 (6.3)
**ASA PS^b^ classification score**														
		1	N/A		N/A		N/A		N/A		N/A		46 (1.4)	
		2	N/A		N/A		N/A		N/A		N/A		1140 (34.4)	
		3	N/A		N/A		N/A		N/A		N/A		2008 (60.6)	
		4	N/A		N/A		N/A		N/A		N/A		112 (3.4)	
		Unknown	N/A		N/A		N/A		N/A		N/A		9 (0.3)	

^a^N/A: not applicable.

^b^ASA PS: American Society of Anesthesiologists Physical Status.

### Analysis Packages and Metrics

**Figure 1:**
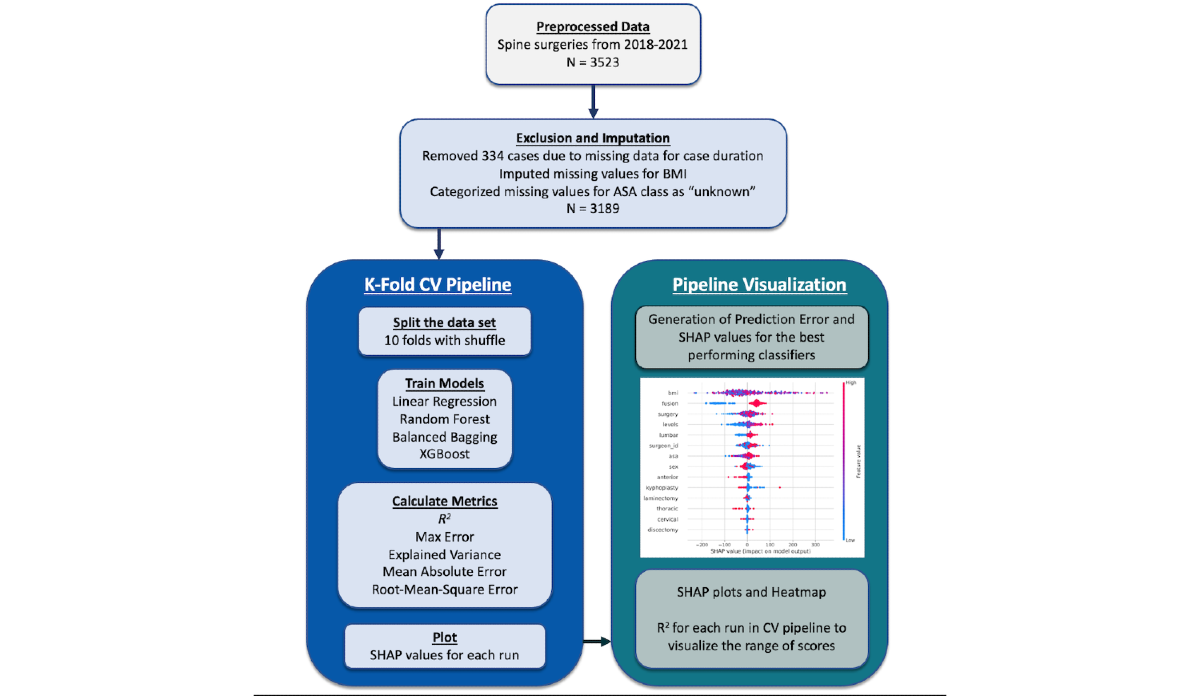
Analysis pipeline-illustration of methodology. ASA: American Society of Anesthesiologists; CV: cross-validation; SHAP: Shapley Additive Explanations.

Python (version 3.7.5; Python Software Foundation) was used for all statistical analyses. The code is provided in the webpage [29]. We calculated the *R*^2^, root-mean-square error (RMSE), mean absolute error (MAE), explained variance, and maximum error for each iteration of k-fold cross-validation (described below) and used those scores to calculate the median scores and plot feature importance using SHAP (Shapley Additive Explanations) and prediction error plots (Figure 1).

### Machine Learning Models

#### Overview

We compared various machine learning-based predictive models to our institution’s conventional model, which predicted case duration using average times (over the last 5 times the surgery was performed by that surgeon) based on the CPT code of the surgery plus adjustments from the surgical attending based on clinical judgment or preference. First, we developed a model using multivariable linear regression. We then evaluated the use of ensemble learning (a process in which multiple models are combined) to calculate a prediction. In this case, we used random forest, bagging, and XGBoost-based regressors (Multimedia Appendix 1). For each model, all features were included as inputs.

#### Multivariable Linear Regression

This is a statistical model that asserts a continuous outcome based on the weighted combination of the underlying independent variables. We tested an L2-penalty-based regression model without specifying individual class weights. This model provides a baseline score and helps make the case for improvement over the evaluation metrics.

#### Random Forest Regressor

Random forest is an ensemble approach (a technique that combines the predictions from multiple machine learning algorithms to make more accurate predictions than any individual model) of decision trees. It is a robust and reliable nonparametric supervised learning algorithm that acts as a means to test further improvements in metrics and determine the feature importance of a data set. The number of tree estimators was set to 1000, the criterion chosen was “squared error,” and the minimum number of samples required to split an internal node was set to 2. All other parameters were left at their default values.

#### Bagging Regressor

Bagging or bootstrap aggregation is another way to build ensemble models. Bagging methods build several estimators on different randomly selected subsets of data. Unlike random forest models, bagging models are not sensitive to the specific data on which they are trained. They would give a similar score even when trained on a subset of the data. Bagging regressors are also generally more immune to overfitting. We built a bagging regressor using the scikit-learn package, where replacement was allowed. The number of estimators was set to 1000 with the base of decision tree regressors, and the samples were drawn with replacement (bootstrap was set to True). All other parameters were left at their default values.

#### XGBoost Regressor

Boosting is another approach to ensemble learning in which decision trees are built sequentially so that each subsequent tree aims to reduce the error from the previous tree. Thus, each subsequent tree learns from previous trees and updates the residual errors. Unlike bagging, boosting uses decision trees with fewer splits; XGBoost is an implementation of a gradient-boosted tree algorithm [30]. We built an XGBoost regressor using the *xgboost* version 1.7.1 package (xgboost developers). The number of estimators used was 1000, the tree method was set to “auto,” and the booster was set to “gbtree.” All other parameters were left at their default values, as described in the documentation of the library.

### Feature Importance

An important function of a model is to uncover potential features that contribute to a given outcome. If a model can predict surgical outcomes efficiently with good specificity, then we can assume that the features of interest that are identified may be relevant and important to the actual surgical outcome. These models can often be opaque with many trees and features of interest, making interpretation of the data difficult. To aid in model interpretation, we used the SHAP model [31]. This module allows for a value to be assigned to each feature used to predict the outcome of a model. Additionally, it provides whether that feature negatively or positively impacts the outcome of that given prediction. If the score is very high or very low, that feature weighs heavily on the model. If the score is close to zero or not well separated, that feature is of lesser importance. Once features are identified and given SHAP values, interpretability is improved because features are concrete and have been assigned importance. Features can then be validated based on scientific rationale and further analysis.

### k-Fold Cross-Validation

To perform a more robust evaluation of our models, we implemented k-fold cross-validation to observe the *R^2^*, MAE, RMSE, explained variance, and maximum error for 10 folds after a shuffle. The data set was first shuffled to account for any sorting and then split into 10 folds, where 1 fold serves as the test set and the remaining 9 sets serve as the training set. This was repeated until all folds had the opportunity to serve as the test set. For each iteration, our performance metrics were calculated on the test set. The median of each performance metric (*R^2^*, RMSE, MAE, explained variance, and maximum error) was calculated thereafter.

## Results

### Overview

There were 3523 spine surgeries identified during this period. After exclusion criteria were applied, 3189 surgeries were included in the final analysis. Among these, there were 39 different kinds of spine surgeries included. The majority of cases involved spinal fusion (n=2433, 76.0%) and were performed in the lumbar region (n=2082, 65.3%). The median ASA PS score was 3, and the majority of patients were male (n=1732, 54.3%; Table 1). The mean of actual surgical case duration among all surgeries was 335.5 (SD 199.9) minutes.

### Performance Evaluation Using Linear Regression

Using all features (Table 1), we developed various machine learning algorithms to predict case duration. The base model, which was the conventional approach against which all machine learning models were compared, was based on our current system’s method to predict surgical times, which is based on the average of the surgical procedures’ case times over the last 5 instances with the ability for the surgeon to change times based on clinical judgment or preference. There was a poor coefficient of determination between the predicted time and actual time based on this approach (*R*^2^=–0.213). We then performed multivariable linear regression trained on 80% of the data and tested on 20% of separate data, which had an *R*^2^ of 0.34. Features that were statistically significant in this model included laminectomy (estimate=218.51, *P*<.001), number of levels performed, ASA PS classification score, and lumbar involvement (estimate=218.51, *P*<.001; Table 2).

**Figure 2:**
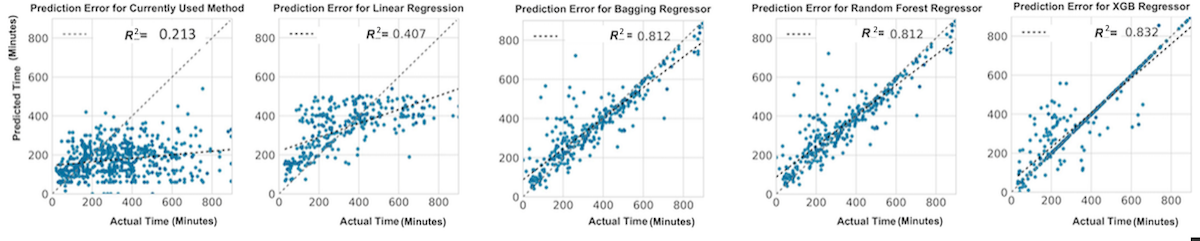
Illustration of the correlation between actual times and predicted surgical times for spine surgery calculated by each model type: predicted times based on procedural averages and surgeon preference or customization, multivariable linear regression, random forest, bagging, and Extreme Gradient Boosting (XGBoost). The data set was split 80:20 (training:test), and the model was trained on the training set and validated on the test set.

Next, we implemented ensemble learning approaches to predicting case duration, in which the models were trained on 80% of the data and tested on a separate 20% of the data. The reason for the 80:20 split was to visualize the *R*^2^ metric for each model (Figure 2). The *R*^2^ metrics for the linear regressor, bagging regressor, random forest regressor, and XGBoost regressor, as well as the currently used method, were 0.407, 0.812, 0.812, 0.832, and 0.213, respectively.

**Table 2:** Results of the multivariable linear regression model predicting actual case duration. We included all features in the model. Because surgical procedure had 39 different procedures, we omitted the values from the table, however, they were included in the model.

			Estimate		SE (minutes)	*P* value
Intercept			–61.89		119.13	.60
**Specific surgical procedure included**						
	Kyphoplasty	–225.43		90.5		.01
	Discectomy	–33.94		94.7		.72
	Fusion	6.17		75.9		.94
	Laminectomy	218.51		41.7		<.001
Anterior approach involved			102.06		93.6	.28
**Number of spine levels involved**						
	1	Reference				
	2	62.57		77.8		.42
	3	–18.15		80.6		.82
	4	74.41		65.3		.25
	5	246.18		61.7		<.001
	6	212.36		106.7		.04
	7	445.13		143.1		.002
**Surgeon performing procedure**						
	A	Reference				
	B	–38.37		13.1		.003
	C	4.32		6.0		.47
**Level of spine involved**						
	Cervical	115.84		53.6		.03
	Thoracic	26.56		34.6		.44
	Lumbar	218.51		52.3		<.001
Male sex			173.34		171.7	.31
BMI (kg/m^2^)			0.38		0.52	.47
**ASA PS^a^ classification score**						
	1	Reference				
	2	66.03		30.1		.32
	3	97.43		25.9		<.001
	4	29.98		30.1		.32
	Unknown	41.97		60.2		.49

^a^ASA PS: American Society of Anesthesiologists Physical Status.

### Median Performance Metrics of Models Using k-Fold Cross-Validation

We calculated various performance metrics for each model by applying a k-fold cross-validation approach and calculated the median scores for each model (Table 3). The linear regression model had an explained variance score of 0.34, an *R*^2^ of 0.40, an RMSE of 162.84 minutes, and an MAE of 127.22 minutes. Among all models, the XGBoost regressor performed the best with an explained variance score of 0.778, an *R*^2^ of 0.77, an RMSE of 92.95 minutes, and an MAE of 44.31 minutes.

**Figure 3:**
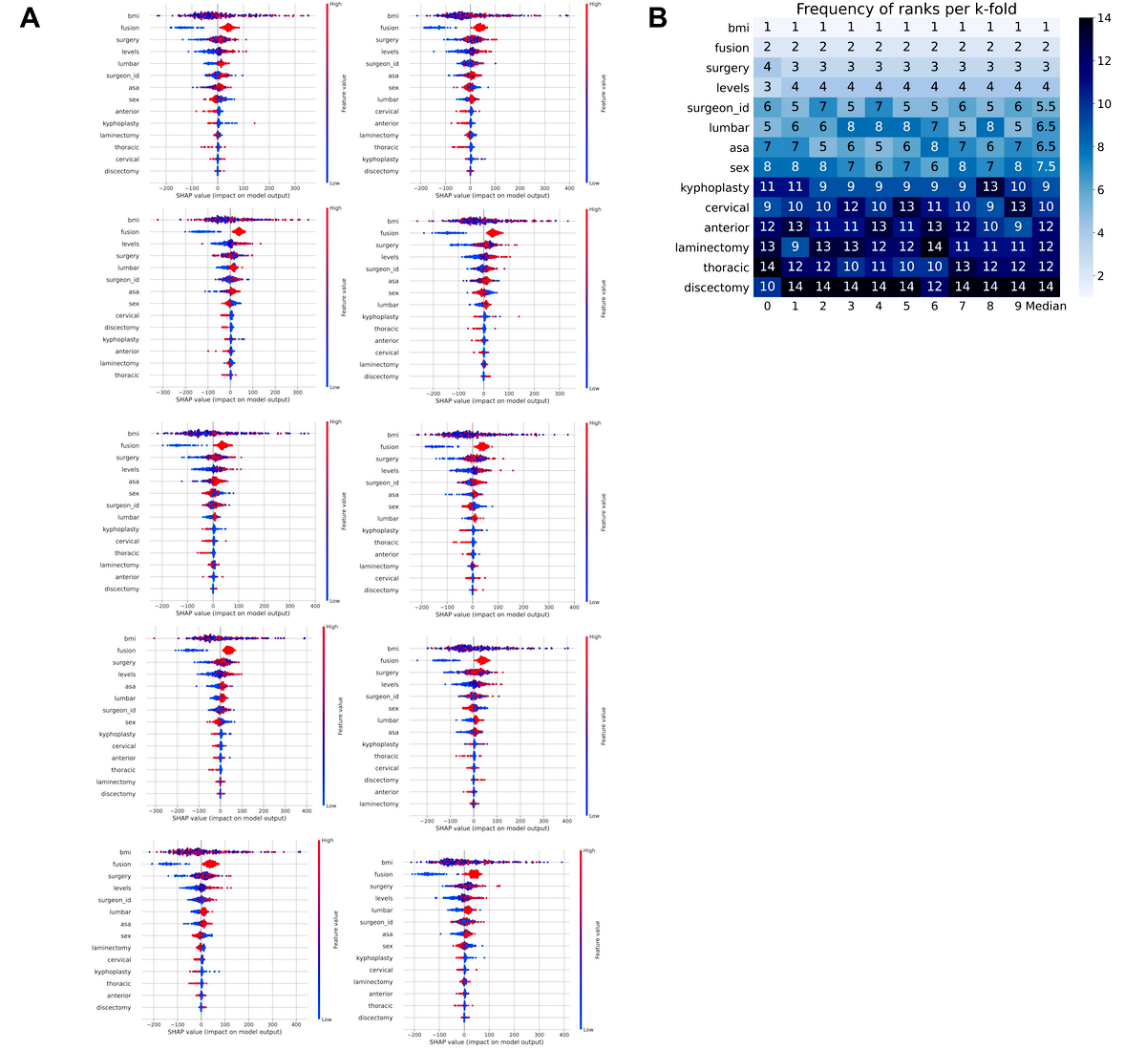
Feature importance from the Extreme Gradient Boosting based on SHAP (Shapley Additive Explanations) values. (A) SHAP analysis for each of the 10 folds; (B) a heat map of the frequency of ranks for each feature per k-fold.

SHAP analysis was performed to describe the features of the XGBoost model with the most impact on model prediction since it was the best-performing model based on the *R*^2^ (Figure 3**)**. Figure 3A illustrates the most important features per fold, whereas Figure 3B illustrates the ranks of each feature’s importance per fold. BMI and spine fusion were consistently the top 2 most impactful features. In order of feature importance, there were then surgical procedure, number of spine levels, operating surgeon, the anatomic location being the lumbar spine, ASA PS classification score, sex, kyphoplasty, the anatomic location being the cervical spine, anterior approach, laminectomy, the anatomic location being the thoracic spine, and discectomy.

**Table 3:** Performance of each machine learning approach predicting case duration of spine surgery. Calculation is based on the median quantified by k-fold cross-validation for the bagging regressor, linear regression, random forest regressor, and XGBoost regressor. Current method is based on average of the last 5 instances of the surgery with surgeons input to modify time.

Model or method	Explained variance	Max error	MAE^a^ (minutes)	RMSE^b^ (minutes)	*R* ^2^
Current method	0.012	847	180.32	243.30	–0.57
Linear regression	0.345	526.29	127.21	162.84	0.34
RF^c^ regressor	0.768	454.59	62.82	96.51	0.76
Bagging regressor	0.769	454.90	62.83	96.51	0.76
XGBoost regressor	0.778	475.72	44.31	92.95	0.77

^a^MAE: mean absolute error.

^b^RMSE: root mean square error.

^c^RF: random forest.

## Discussion

### Principal Findings

We found that the use of ensemble learning with the patient and procedural-specific features (variables that are known preoperatively and attainable from the electronic medical record system) outperformed the prediction of spine surgery case duration when compared to models that use historic averages and surgeon preferences. Unique to our approach of predicting surgical time for this heterogenous surgical population was the granularity of features (eg, patient and surgical characteristics) combined with an ensemble learning approach. The reference model (the time estimated based on historic averages and surgeon preference) had poor performance. We then implemented machine learning-based models using features including procedural details (ie, number of spine levels, patient positioning, surgeon, level of spine region involved, etc) and patient-specific details (ie, body mass index, sex, ASA score, etc) and demonstrated improved performance. While linear regression improved *R^2^* to 0.34, the use of XGBoost, random forest, and bagging improved it further (0.77, 0.71, and 0.71, respectively). Such models could be relatively easy to integrate into a resource-capable electronic medical record system, given that the included features could be obtained automatically from the electronic record preoperatively.

The usage of historic averages or CPT code-based estimations for spine surgery scheduling may be inaccurate given that some determinants of case duration may not be accounted for in the prediction. These features include surgeon experience, level of the spine region involved, number of levels, type of surgery (ie, kyphoplasty, fusion, laminectomy, etc), need for multiple surgeries, patient positioning, and patient body mass index. The inclusion of these features into our models results in a substantial improvement in prediction accuracy. Accurate prediction of operation times has long been discussed as a means to improve operating room efficiency and patient care [14]. Recent implementations of such models have demonstrated these improvements across a variety of measures. A recent randomized clinical trial found that a machine-learning approach increased prediction accuracy, decreased start-time delay, and decreased total patient wait time [32]. A similar randomized controlled trial demonstrated increased throughput and decreased staff burnout [32,33]. Subsequently, decreases in delays and wait times result in lower costs and increased caseloads, which can further drive cost-effectiveness [34,35]. Associations between wait times and postoperative complications provide evidence that proper identification and mitigation of delays can improve outcomes as well [36]. Overall, improvements in patient scheduling, case duration, and staffing may result in enhanced efficiency and potentially superior patient outcomes. Understanding and identifying the features that are key in lessening the burden of misused surgical time is crucial with the trending increases in caseload burden and impacted hospital resources.

Ensemble learning essentially uses an “ensemble” of predictive models and calculates the overall prediction based on the individual predictions from each model within the “ensemble.” In this case, we leveraged ensemble learning using decision tree-based machine learning algorithms: random forest, bagging, and boosting. Our results demonstrated a substantial improvement with XGBoost compared to the other ensemble approaches as well as linear regression. XGBoost often performs better than random forest because it prunes nodes if the gain of a node is minimal to the model [30]. Random forest generates the tree to a greater depth because it does not prune nodes and relies on a majority vote for the outcome. This can result in overfitting in random forest models. Random forest may also give preference to classes that are associated with categorical variables, which do not occur in XGBoost. Because XGBoost is an iterative process, it gives preference to features that enable the regressor to predict low-participation classes. Additionally, XGBoost is more efficient with the unbalanced data sets often seen in medical or biological data. Alternatives such as linear regression work well when the data is straightforward and well-distributed. The more complex the data set, the better a bagging or tree-based model will work. With ensemble approaches, nonlinear relationships between features may be captured, and a “strong” model is developed based on learning from “weaker” models, in which residual errors are improved. Thus, the use of ensemble learning in this clinical scenario—where there is a complex interplay between features—may be superior to a statistical approach that only models linear relationships (ie, linear regression). Future studies may benefit from other approaches such as support vector machines, which could be implemented to focus on accuracy, or penalized regressors, which could provide increased interpretability.

Oftentimes, machine learning approaches are described as “black boxes” because the interpretation of the importance of features to the predictive model is challenging. The implementation of an explainer model such as SHAP values is one way to elucidate the importance of features. In this study, SHAP identified that BMI is the most important feature of the model and provides weight and context to the feature about the other identified features [37,38]. BMI may be associated with increased case duration due to the additional technical and positioning challenges. Sex was also identified as an important feature. This finding is congruent with current research that demonstrates women are more likely to have bone loss earlier than men, and bone loss has been shown to affect surgical outcomes and recovery due to poor bone remodeling and healing [39,40]. Other interesting features with an important impact included the operating surgeons themselves, the ASA PS classification score, and the number of spine levels operated. It makes sense to include surgeons as a feature in predictive modeling as each physician may have different styles and comfort levels that could impact surgical time. The ASA PS classification score represents a patient’s comorbidity burden and could suggest that patients with a higher comorbidity burden would require longer anesthesia times. Finally, it makes sense that the number of spine levels contributes to case duration, as this has a potentially linear relationship to how long surgery would take. Being able to put various features into the context of the research question is essential for translating the findings into actionable metrics. Overall, the SHAP analysis identified clinically relevant features for future exploration and evaluation.

There are several limitations to the study, mainly its retrospective nature; thus, the collection and accuracy of the data are only as reliable as what is recorded in the electronic medical record system. The current institutional practice for estimating scheduled case duration was based on the historic averages of the last 5 surgeries, with the surgeon’s ability to change the times based on clinical judgment or preference. We do not have data on why and when surgeons changed the times. In addition, there were some missing data for actual case duration, but this only led to the removal of 5.9% of the initial data set. There may also be several features not included in the models that may substantially contribute to time estimates, including surgical resident involvement (and their level of training) or surgical instruments used. Furthermore, there are other machine learning algorithms that we did not test, including support vector machines and penalized regressors. Despite these limitations, we were able to develop a predictive model using XGBoost with a high *R*^2^ value (>0.7). These findings would need to be validated externally and prospectively to determine their generalizability to spine surgeries.

### Conclusions

Operating room efficiency is a key factor in maintaining and growing institutional profits. Additionally, improvements in operating room efficiency contribute to enhanced patient care and satisfaction. Given the technical and anatomical heterogeneity in spine surgeries, it has been a challenge to predict case duration using conventional methods at our institution. This method can be applied in the future to standard and heterogenous surgical procedures with or without class imbalance to identify key obstacles to future surgical efficiency; however, it is crucial to develop robust models to more accurately predict schedule case length. In our study, we demonstrated that patient and surgical features that are easy to collect from the electronic medical record can improve the estimation of surgical times using machine learning-based predictive models. Future implementation of machine learning-based models presents an alternative pathway to use electronic medical record data to advance surgical efficiency and enrich patient outcomes.

## Appendix

Multimedia Appendix 1 Illustration of how each ensemble learning algorithm computes the prediction: (A) random forest, (B) bagging, and (C) Extreme Gradient Boosting (XGBoost).

## Abbreviations

ASA PS: American Society of Anesthesiologists Physical Status
CPT: Current Procedural Terminology
MAE: mean absolute error
RMSE: root-mean-square error
SHAP: Shapley Additive Explanations
XGBoost: Extreme Gradient Boosting
