# Supplementary figures and images for: An Ensemble Learning Approach to Improving Prediction of Case Duration for Spine Surgery: Algorithm Development and Validation
Source: JMIR Perioper Med. 2023 Jan 26;6:e39650. doi: 10.2196/39650 (PMC9912154; doi:10.2196/39650)

# A Random Forest

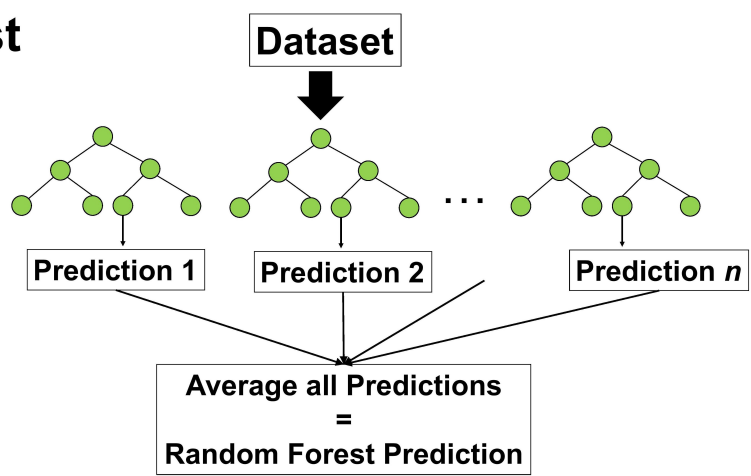

# B Bagging

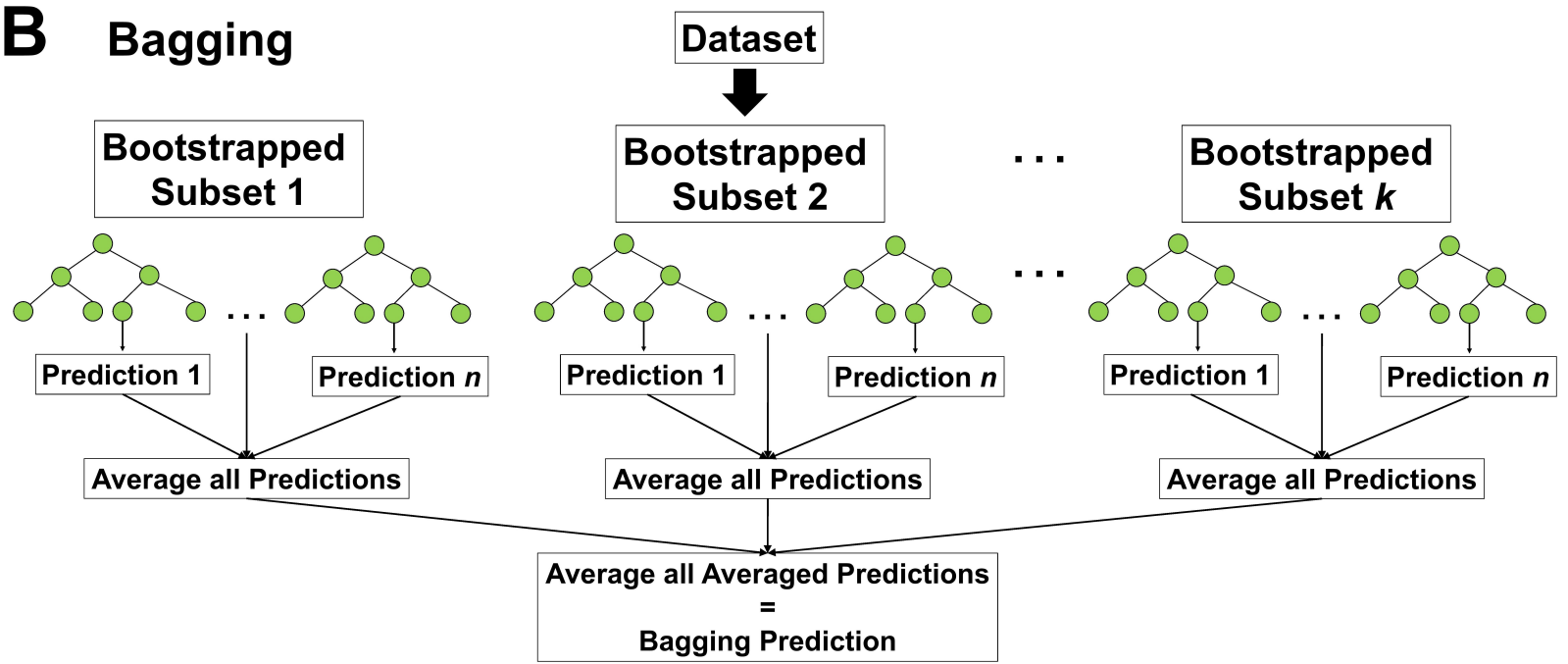

# C XGBoost

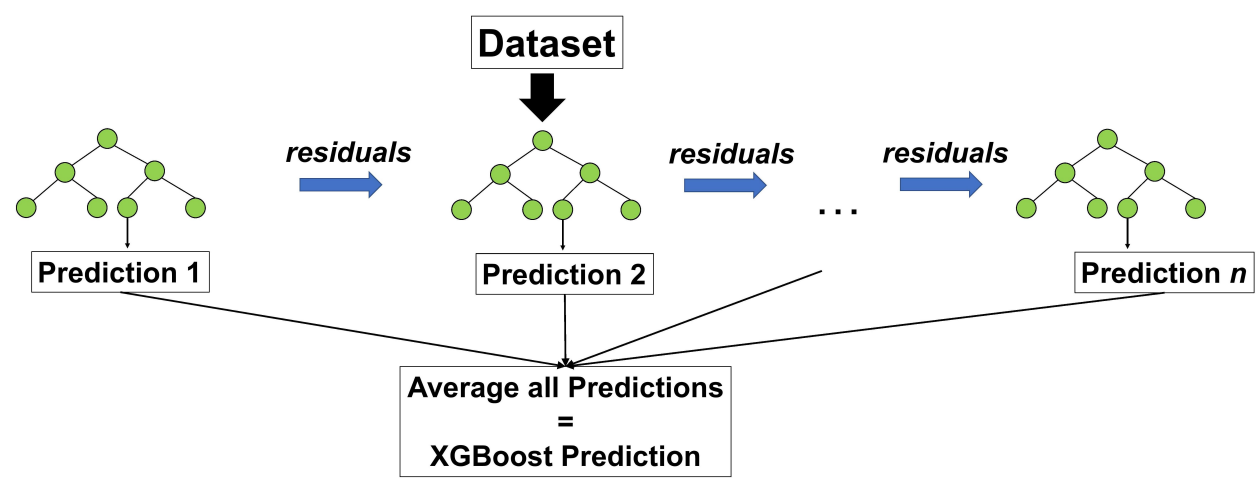

Supplement: Multimedia Appendix 1 [file periop_v6i1e39650_app1.pdf]
